# Morphological correlates of injury-induced reorganization in primate somatosensory cortex

**DOI:** 10.1186/1471-2202-5-43

**Published:** 2004-11-08

**Authors:** James D Churchill, Jason A Tharp, Cara L Wellman, Dale R Sengelaub, Preston E Garraghty

**Affiliations:** 1 Department of Psychology, Saint Louis University, St. Louis, Missouri 63103, USA; 2 Department of Psychology, Program in Neural Science, Indiana University, Bloomington, Indiana 47405, USA

## Abstract

**Background:**

Topographic reorganization of central maps following peripheral nerve injury has been well characterized. Despite extensive documentation of these physiological changes, the underlying anatomical correlates have yet to be fully explored. In this study, we used Golgi impregnation and light microscopy to assess dendritic morphology following denervation of the glabrous hand surface in adult primates.

**Results:**

After survival durations that permit complete physiologically-defined reorganization, we find a systematic change in the dendritic arborization pattern of both layer II/III pyramidal and layer IV spiny stellate cells in the contralateral hand region of area 3b, compared to unaffected cortical areas. In general, our analyses indicate a progressive expansion of distal regions of the dendritic arbor with no appreciable changes proximally. This pattern of distal dendritic elaboration occurs for both basilar and apical dendrites.

**Conclusions:**

These observations are consistent with the notion that latent inputs gain expression in reorganized cortex after nerve injury via their influence through contacts with more distally located termination sites.

## Background

The ability of the nervous system to modify its output in accordance with experiential demands is a central tenet of neuronal plasticity. For many years, the view of critical periods permeated our beliefs; almost dictating that plasticity beyond such epochs was, at best, minimal. The seminal experiments of Merzenich, Kaas and colleagues [1,2] have proved instrumental in moving the field beyond this restrictive mindset by showing that the central representation of the skin surface is subject to dramatic modification following peripheral nerve injury in adult primates. On the foundation of these observations, great strides have been made in understanding the mechanisms [3-9] and extent [10-13] of this phenomenon. These findings have generalized beyond sensory systems and collectively have been interpreted as reflective of fundamental properties of the nervous system.

While physiological techniques are frequently used to characterize topographic (re)organization of central maps, the underlying anatomical correlates have not been thoroughly investigated. Using intracellular injection techniques, thalamic axons have been reported to innervate a much broader sector of cortex than necessary to represent typical receptive field size, suggesting the existence of "latent" inputs [14,15]. Disinhibition is a strong candidate as the primary mechanism during the immediate phase of somatotopic reorganization following nerve injury [16-18]. While unmasking of latent inputs may account for a portion of the overall reorganization [19], modification of central maps is neither complete immediately following nerve injury [1,20-22] nor dependent on a single mechanism [20,22-25]. Moreover, topographic reorganization appears to be permanent in nature [26], while at least some neurochemical changes have shown to be relatively transient [6,18]. Together, these observations suggest that alterations in the underlying anatomical connectivity might provide a stable platform for the maintenance of modified somatotopy.

In this study, we report our examination of neurons in two cortical layers; spiny stellate cells in layer IV, as this is the primary input target of thalamocortical axons; and pyramidal neurons in layer II/III, as supragranular changes have been shown to precede somatotopic modification in the granular cell layer [27]. We predicted that dendritic arborization in the affected areas would be altered following peripheral nerve injury, providing an anatomical correlate of the functional changes. If the anatomical correlates of physiologically-defined changes can be readily observed, our understanding of the mechanisms underlying such changes would be greatly enhanced.

## Results

**Figure 1:**
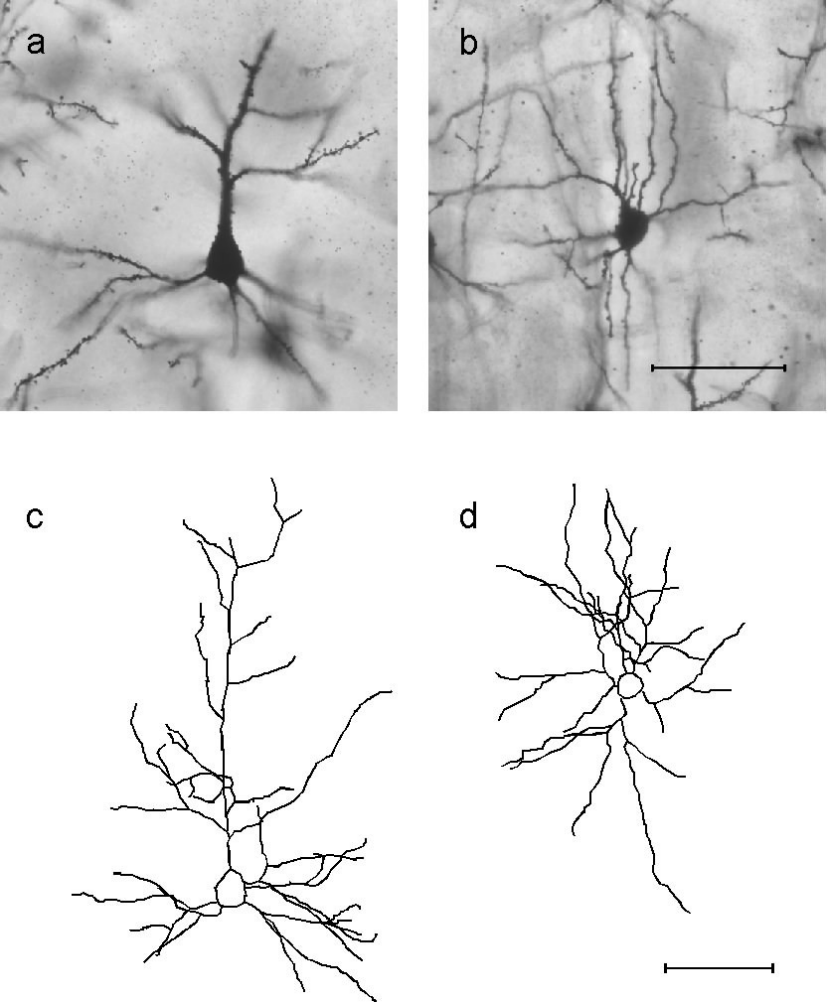
**Photomicrographs and reconstructions of Golgi-filled neurons**. **a**: A typical layer II/III pyramidal cell used in the analysis of dendritic arborization. **b**: A typical layer IV spiny stellate cell. **c,d**: Reconstructions of a pyramidal and a stellate cell. Scale bar = 50 μm.

Figure 1 presents typical Golgi-filled layer II/III pyramidal (Fig. 1A) and layer IV spiny stellate cells (Fig. 1B). Figures 1C (pyramidal) and 1D (stellate) are corresponding reconstructions of the same two cell types. Our initial inspection of the data revealed considerable heterogeneity as one moved from proximal to distal regions of the dendritic arbor. With regards to the proximal halves of the arbors, we observed no statistically significant differences between deprived and control groups for either basilar or apical dendrites.

### Layer II/III pyramidal neurons

**Figure 2:**
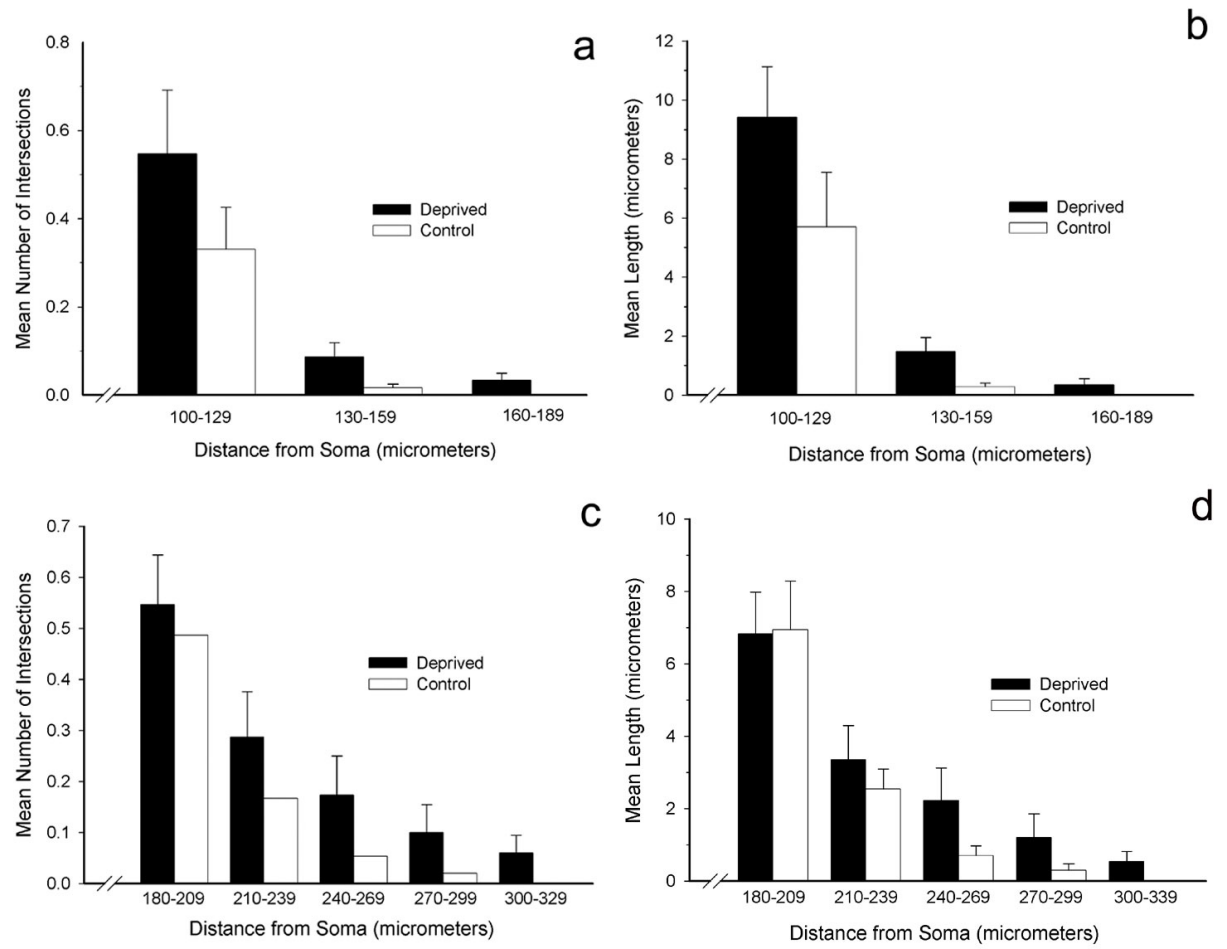
**Dendritic arborization pattern in the distal sectors of layer II/III pyramidal cells**. The figure depicts the extent of arborization as a function of distance from the cell body using a Sholl ring analysis. On average, basilar dendrites in deprived cortex are both more complex (increased number of intersections; Fig 2a) and longer (dendritic length; Fig 2b) than those from controls. Likewise, apical dendrites in deprived cortex are also more complex (intersections; Fig 2c) and longer (length; Fig 2d), compared to controls. All effects were statistically significant.

We found a greater number of intersections in the distal halves of dendritic arbors of pyramidal cells in deprived relative to control cortex. For basilar dendrites, there is a 92.0% increase in the number of intersections in the distal arbors of deprived cells relative to control cells (9/9, p < .01; see Fig. 2a). Likewise, we find an 89.5% increase in total basilar dendritic length in the distal sectors of deprived cortical pyramidal cells, compared to control neurons (9/9, p < .01; see Fig. 2b). For apical dendrites, there are 63.1% more intersections in distal portions of the arbors of deprived cells relative to controls (15/16, p < .01; see Fig. 2c). Finally, the average length of the distal apical dendrites of deprived pyramidal cells is 37.4% greater than in controls (14/16, p < .01; see Fig. 2d).

### Layer IV spiny stellate cells

**Figure 3:**
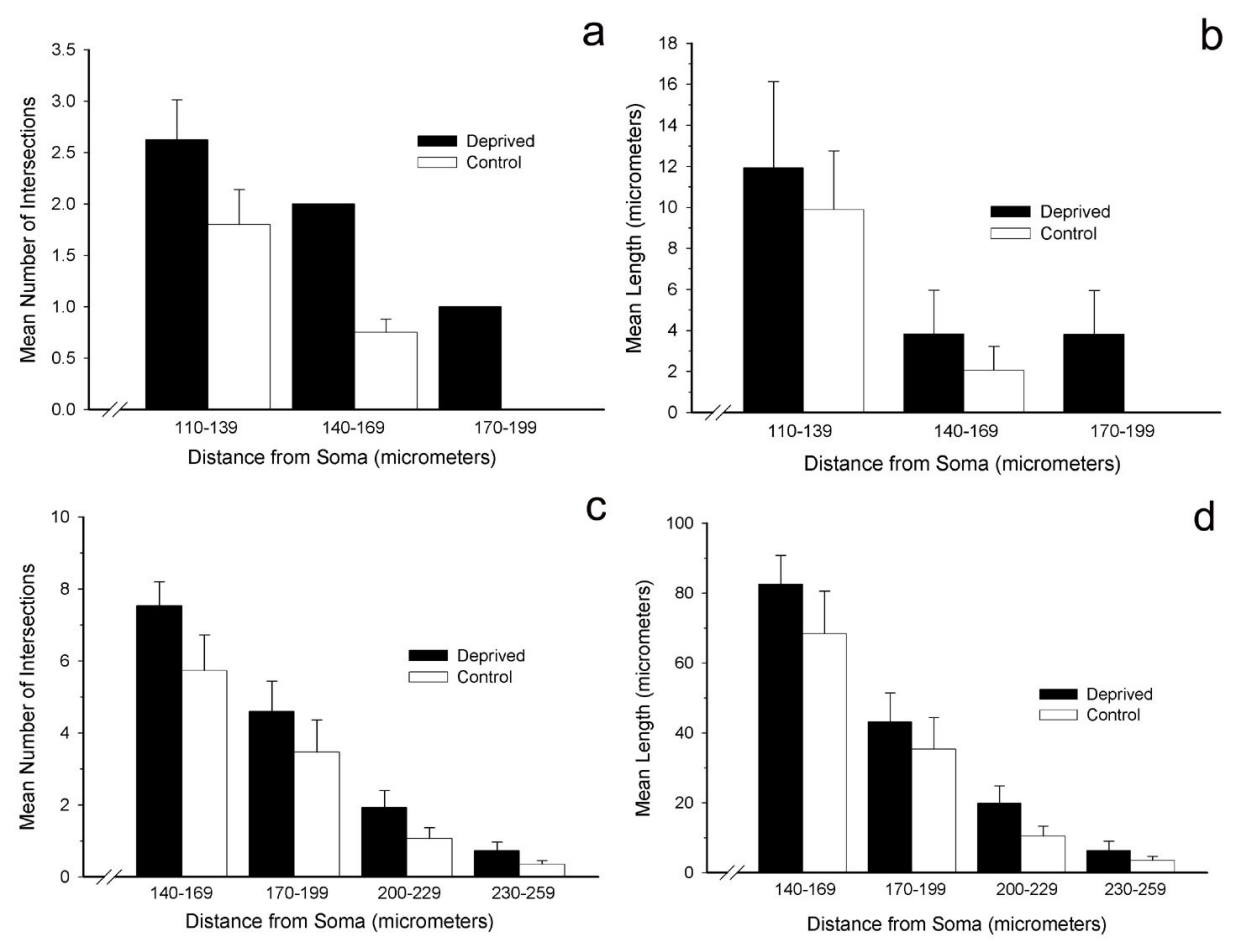
**Dendritic arborization pattern in the distal sectors of layer II/III stellate cells**. The figure depicts the extent of arborization as a function of distance from the cell body using a Sholl ring analysis. On average, basilar dendrites in deprived cortex are both more complex (increased number of intersections; Fig 3a) and longer (dendritic length; Fig 3b) than those from controls. Likewise, apical dendrites in deprived cortex are also more complex (intersections; Fig 3c) and longer (length; Fig 3d), compared to controls. All effects were statistically significant.

A largely comparable set of outcomes were found for spiny stellate cells in layer IV. For the distal sectors of basilar dendrites, there are 66.7% more intersections in arbors of deprived relative to control neurons (9/9, p < .01; see Fig. 3a). Similarly, the overall average length of distal basilar dendrites is 92.4% longer in deprived stellate cells compared to controls (9/9, p < .01; see Fig. 3b). For the distal apical dendritic arbors, deprived stellate cells have, on average, 25.5% more intersections than are found in control neurons (11/12, p < .01; see Fig. 3c). Conversely, while the distal apical dendrites of deprived neurons are 20.5% longer than controls, on average, this difference is not statistically significant (8/12, p > .10; see Fig. 3d).

### Differential effects of deprivation on basilar versus apical dendrites

**Figure 4:**
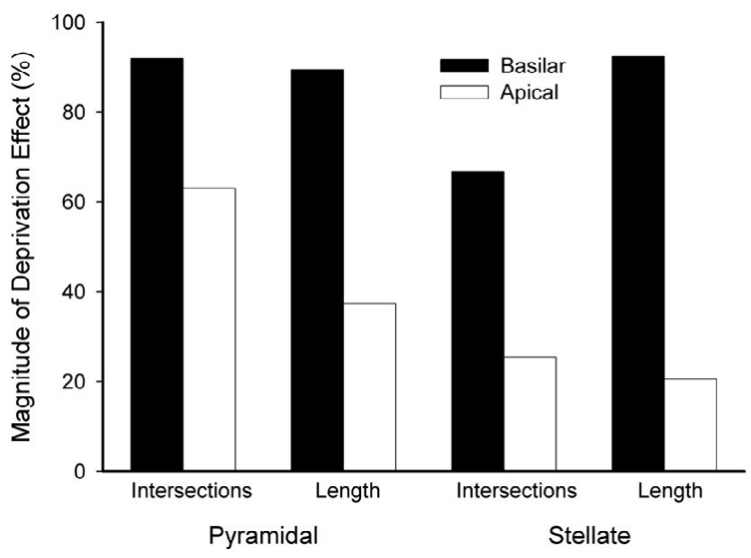
**Comparison of basilar vs. apical effects for all cells analyzed**. The figure illustrates that for both cell types (pyramidal and stellate) and metric of dendritic arborization (intersections and length), the magnitude of the effect was reliably larger for basilar dendrites than for apical dendrites.

Deprivation of a specific region of somatosensory cortex by nerve transection clearly had a detectable effect on the distal portions of both the basilar and apical dendrites of both layer II/III pyramidal cells and layer IV spiny stellate cells. The data also support the contention that the basilar dendrites of both cell types were more profoundly affected by deprivation than were their apical dendrites. The magnitudes of the deprivation effects are more pronounced in basilar than in apical dendrites (see Fig. 4; Mann-Whitney = 0; p < .01).

## Discussion

### General observations

In the present experiments, we investigated whether changes in dendritic morphology of neurons in deprived somatosensory cortex are correlated with the well-documented topographic reorganization that follows peripheral nerve injury in adult primates. Our general findings were that in deprived cortical areas, dendritic arbors were expanded distally, while being unaffected proximally. This pattern was found for both the basilar and apical dendrites of layer IV spiny stellate and layer II/III pyramidal neurons, though the effects were more pronounced for the basilar dendritic arbors, a difference that is consistent with previous reports [28]. Measures of dendritic length and the frequency of intersections, both well accepted metrics of dendritic arborization, yielded generally similar patterns of observations. These anatomical changes could well provide the means by which the functional changes in map topography proceed. Alternatively, they could reflect generic neural responses to deprivation *per se*, and have little or nothing to do with the functional reorganization.

### Does nerve injury-induced reorganization reflect the strengthening of normally latent inputs?

Previous research has shown that the spread of thalamocortical arbors is much broader than necessary for the expression of typical receptive field size in primary somatosensory cortex [14,15]. Because of this disparity between the grain of the cortical topographic map [29] and the far more extensive thalamocortical anatomy, we have suggested that all parts of thalamocortical arbors cannot be equally effective in conveying suprathreshold receptive field information to the cortex, and that changes in synaptic efficacy could sustain the topographic plasticity that follows peripheral nerve injury [14,30]. Our observations of subthreshold, latent inputs to the cortex [3], and the emergence of their expression when dominant inputs are attenuated [31] lend support for this idea. Moreover, these presumptive latent inputs are largely prevented from gaining expression in cortex when NMDA glutamatergic receptors are blocked [20,23]. Such a blockade could prevent reorganization by preventing changes in the strength of existing synapses [e.g., [32]], by interfering with neurite outgrowth [33], or both. In any event, such latent inputs become evident only when the normally expressed, dominant inputs are somehow weakened – via pharmacological disinhibition [16,18,34], nerve injury [19,35], or use-dependency [36-38]. The data reported here are consistent with this notion, and suggest that distal sectors of the dendritic arbor may be selectively innervated by these latent inputs.

Our observations that distal regions of apical dendrites, which clearly reside in upper cortical layers, are modified come as no surprise. Previous work has shown that layer IV spiny stellate cells act primarily as intracolumnar signal processors; while pyramidal cells integrate both horizontal and top-down information [39]. Supragranular layers appear to be particularly fertile to altered stimulation patterns as cortical reorganization occurs initially in the outermost layers of cortex, followed later by changes in the granular cell layer [27]. Measures of astrocytic recruitment mirror this outside-to-inside temporal progression of experience-dependent reorganization as well [40]. The selective elaboration of distal regions of the dendritic arbor is also consistent with data that implicate intracortical pathways as playing a major role in cortical reorganization [41,42], though, clearly, the contribution of bottom-up processes cannot be discounted [12].

### Is reorganization a secondary consequence of other mechanisms/processes?

While morphological changes may be less likely to account for acute changes in somatotopy after nerve injury, restructuring of the underlying anatomy could well correlate with the longer-term, persistent changes in cortical topographic maps. The modifications of distal dendritic regions reported here may be interpreted from at least three possible, non-exclusive, perspectives. First, expansion of the distal arbor may be a homeostatic response to a reduction in stimulation frequency/pattern following nerve injury. Progressive elaboration of the distal arbor might be an attempt to maintain optimal stimulation levels, and, thus, normal interneuronal trophic relationships. The altered somatotopy could be construed as simply the epiphenomenonal consequence of the activation of a homeostatic response. Second, the elaboration of the distal arbor may be a general property of the nervous system, a mechanism that permits the brain to respond in a dynamic and adaptable manner [43]. This supposes that the functional changes in cortex that follow nerve injury are adaptive, and that has not been convincingly demonstrated. Third, the observation that changes occur distally may simply reflect the fact that areas relatively distant to the soma are more vulnerable/susceptible to changes, regardless of the adaptability of such changes [44]. The reliability of progressively distal changes independent of dendritic location (apical or basilar) is certainly consistent with this idea. While these possibilities are not mutually exclusive, and certainly not all-inclusive, we believe that expansion of the distal arbor reported here is reflective of the altered activation pattern following nerve injury and serves as a long-term trace of this modified stimulation pattern.

## Conclusions

Considering the range of survival durations following nerve injury in the current study, the observation of modifications to both layer IV spiny stellate and layer II/III pyramidal neurons was not unexpected. While this broad survival range may have "smeared" our snapshot with respect to the temporal integration of anatomical changes, our intention was simply to determine whether morphological changes were occurring at any point during the reorganization process. Our data clearly indicate that the anatomy in affected cortical areas is subject to modification and that the morphological changes observed may be related to the functional reorganization revealed electrophysiologically. We have begun experiments to better refine the temporal window in which these changes become evident.

In sum, we have shown that just as the functional responsiveness of the mature primate nervous system is susceptible to change, so is the underlying anatomy. Our observations that the anatomical changes appear to be either potentiated in, or possibly restricted to, distal regions of the dendritic arbor provide additional insight into the mechanisms involved in the physiological changes. Further research will be instrumental in determining the exact role that the underlying anatomy plays in this complex reorganization process.

## Methods

Adult squirrel monkeys (*Saimiri scireus *or *Saimiri bolivensius*) were socially housed with food and water available *ad libitum*. In six animals, the median and ulnar nerves to one hand were transected following the principles of animal care detailed in NIH publication no. 86–23. The local institutional animal care and use committee approved all procedures prior to initiation of any experiments. Briefly, monkeys were anesthetized with an intramuscular injection of a mixture of ketamine hydrochloride (25–30 mg/kg) and xylazine (0.5–1.0 mg/kg). Their forearms were shaved and prepared for surgery with alternate scrubbings of povidone-iodine and alcohol. Under sterile conditions an incision was made along the midline of the ventral forearm, the median and ulnar nerves were located by blunt dissection and cut about midway between the elbow and wrist. The epineural sheath of the proximal stump was retracted 0.5–1.0 cm, and the exposed nerve avulsed. The empty epineural sheath was re-extended, folded back upon itself and ligated. The nerve stumps were repositioned and the incision closed with sutures. Post-surgically, all subjects received penicillin, dopram hydrochloride, and dexamethasone injections. Subjects were permitted to recover for a period of time previously shown sufficient to permit complete reorganization of the hand representation in cortical area 3b (3–52 months, mean = 15.3). Two additional subjects served as naïve controls.

Following electrophysiological mapping of the affected cortical areas, animals were overdosed and perfused transcardially with 0.9% saline. Brains were extracted and immersed in a modified Golgi–Cox solution for 11 days, thereafter dehydrated and embedded in celloidin. Tissue blocks were sectioned coronally at 150 μm in thickness for morphological assessment and every fourth section in the series was cut at a thickness of 90 μm for Nissl staining to facilitate cytoarchitectonic identification of area 3b borders. Free-floating sections were processed and mounted on glass, according to previously reported procedures [45].

Analysis of dendritic morphology was conducted blind to experimental condition on thoroughly impregnated cells using methods described by Sholl [46]. For each animal, ten layer IV spiny stellate and ten layer II/III pyramidal cells from the hand area of somatosensory area 3b contralateral to the nerve injury were drawn using Neurolucida (MicroBrightfield) at 600 × magnification. In addition, ten area 3b cells of the same two types located outside of the hand representation were drawn to serve as controls.

We have shown previously that dominant and latent inputs from the three nerves innervating the hand have overlapping territories in area 3b [13,32], with the latent inputs gaining expression when the dominant inputs are weakened with peripheral nerve transection. These observations prompted us to treat the proximal and distal portions of the dendritic arbors separately in our statistical comparisons. To accomplish this goal, we divided the arbors into proximal and distal halves using the Sholl ring halfway between the soma and the most distal dendritic process as the dividing point. For dendritic length and intersection comparisons, the deprived and control averages for each Sholl ring were compared. A simple binomial test [47] was then applied to determine whether a systematic, statistically significant difference exists between those sets of means.

## Authors' contributions

JDC participated in design of study, conducted the histological processing, drafted the manuscript: JAT conducted some of the histological processing: CLW participated in design of study: DRS participated in design of study: PEG participated in design of study, conducted statistical analyses, conceived and coordinated the study. All authors read and approved the final manuscript.
